# Ambient Music Decreases EEG Burst-Suppression Ratio During General Anesthesia in Rats

**DOI:** 10.3390/s26113527

**Published:** 2026-06-02

**Authors:** Vlad-Petru Morozan, Mihai Stancu, Alexandru-Cătălin Pâslaru, Bogdan Pavel, Alexandra Mocanu, Alexandru Călin, Leon Zăgrean, Ana-Maria Zăgrean, Mihai Moldovan

**Affiliations:** 1 Division of Physiology—Neuroscience, Carol Davila University of Medicine and Pharmacy, 050474 Bucharest, Romania; vlad-petru.morozan@drd.umfcd.ro (V.-P.M.); stancu@bio.lmu.de (M.S.); catalin.paslaru@umfcd.ro (A.-C.P.); bogdan.pavel@umfcd.ro (B.P.); alexandra.mocanu2@s.unibuc.ro (A.M.); alexandru.calin@kcl.ac.uk (A.C.); leon.zagrean@umfcd.ro (L.Z.); ana-maria.zagrean@umfcd.ro (A.-M.Z.); 2 Department of Plastic and Reconstructive Microsurgery, Dr. Carol Davila Central Military Emergency University Hospital, 010825 Bucharest, Romania; 3 Division of Neurobiology, Faculty of Biology, Ludwig Maximilian University of Munich, 82152 Planegg-Martinsried, Germany; 4 Victor Gomoiu Children’s Hospital, 022102 Bucharest, Romania; 5 Doctoral School of Philosophy, Faculty of Philosophy, University of Bucharest, 060024 Bucharest, Romania; 6 Department of Basic and Clinical Neuroscience, Institute of Psychiatry, Psychology and Neuroscience, The Maurice Wohl Clinical Neuroscience Institute, King’s College London, Cutcombe Road, London SE5 9RT, UK; 7 Department of Clinical Neurophysiology, King’s College Hospital NHS Foundation Trust, Denmark Hill, London SE5 9RS, UK; 8 Department of Neurology, North Zealand Hospital, 3400 Hillerød, Denmark; 9 Department of Neuroscience, University of Copenhagen, 2200 Copenhagen, Denmark; 10 Department of Clinical Neurophysiology, Rigshospitalet, 2100 Copenhagen, Denmark

**Keywords:** EEG, general anesthesia, burst-suppression reactivity, auditory, photic, intensive care neuromonitoring

## Abstract

**Highlights:**

**What are the main findings?**

- In rats, ambient sound stimulation transiently decreased suppression ratio during targeted burst-suppression across different anesthetics.
- The reduction in suppression ratio (reactivity) to auditory stimulation was associated with an increased bursting rate comparable to photic stimulation.

**What are the implications of the main findings?**

- Targeted suppression ratio reflects both anesthetic depth and the level of ambient stimulation.
- Ambient sound in the operating theater or intensive care settings could influence burst-suppression monitoring.

**Abstract:**

During deep anesthesia, the EEG becomes discontinuous. Burst-suppression is often an intended target during deep sedation or medically induced coma. Within this state, anesthetic depth is commonly monitored by the suppression ratio (SR), which expresses the fraction of time spent in suppression. However, accumulating evidence suggests that SR remains reactive to external stimulation. We tested whether ambient music commonly played in operating theaters alters the SR in male Wistar rats under sevoflurane, chloral hydrate, or isoflurane anesthesia. To this end, the first 60 s of the Stayin’ Alive audio track by the Bee Gees were played to examine auditory-induced burst-suppression reactivity in an experimental model previously established for intermittent photic stimulation. SR and the burst-suppression reactivity index (BSRi, derived as the decrease in SR during stimulation normalized to pre-stimulation SR) were measured in repeated trials. Auditory stimulation transiently decreased SR under all three anesthetics. This was associated with an increase in the rate of burst occurrence without increased burst duration. The BSRi changes depended on the anesthetic type, comparable to photic stimulation. Our experimental data suggest that the suppression ratio used to monitor targeted burst-suppression reflects both anesthetic depth and the level of ambient stimulation. Ambient sound in the operating theater or intensive care settings could influence EEG-based measures used for anesthesia monitoring.

## 1. Introduction

During deep anesthesia, the electroencephalogram (EEG) may become discontinuous, with intermittent high-amplitude bursts of activity against a suppressed background [1,2,3]. Burst-suppression is often the intended target for achieving deep anesthesia or medically induced coma. Within burst-suppression, anesthetic depth is commonly monitored by the suppression ratio (SR), which expresses the fraction of time spent in suppression. SR is also incorporated into EEG monitors, such as the Bispectral Index (BIS) [4,5,6,7].

The assessment of SR depends on several technical details, including how bursts and suppressions are defined, recording geometry, and the computational process used to derive a final metric [1,2,3,8]. Both experimental and clinical work have shown that external stimulation can trigger or facilitate cortical bursting activity during burst-suppression [2,3,9,10,11]. For this reason, SR could reflect not only anesthetic level, but also residual sensory responsiveness [1,2,3].

A decrease in SR (reactivity) due to peripheral nerve stimulation was shown in patients with healthy brains during intraoperative evoked potential monitoring [10], although this was a special setting. In the operating room or in busy intensive care environments, background noise, alarms, conversation, instruments, and music are far more common forms of ambient stimulation [12,13]. Auditory evoked potentials have long been studied as indicators of anesthetic state in humans [14], and rat studies have shown that auditory evoked EEG responses are retained under anesthesia and are influenced by the anesthetic conditions [15]. Nevertheless, the level of ambient auditory stimulation is difficult to standardize in the clinical setting.

To assess whether ambient sound could influence EEG-based measures used for anesthesia monitoring, our study aimed to experimentally assess the effect of ambient auditory stimulation on SR during targeted anesthetic burst-suppression. We used an in vivo rat EEG model which showed reproducible SR-based reactivity to intermittent photic stimulation [16]. For auditory stimulation we played the first minute of Stayin’ Alive by the Bee Gees (RSO, 1977, London, UK), because a published survey identified this song as surgeons’ favorite in operating theaters [12]. Specifically, the study addresses three linked questions: (1) Does ambient sound alter the SR? (2) What is the mechanism underlying the SR changes? (3) How do the auditory reactivity changes compare with photic reactivity in this model?

The study was deliberately framed around suppression ratio rather than alternative signal-processing endpoints. This choice reflects the translational question addressed here: whether an environmental auditory stimulus can confound the type of suppression-based metric currently used in experimental and clinical anesthesia monitoring.

## 2. Materials and Methods

### 2.1. Experimental Design

EEG recordings were obtained from adult male Wistar rats under deep general anesthesia induced by either sevoflurane, chloral hydrate, or isoflurane. The rats were randomized to receive auditory stimulation (AUD) or photic stimulation (PHOT). Two rats were initially studied for each anesthetic group and each stimulation modality. Additional animals were added when needed to reach at least 20 valid stimulation trials per group (Table 1). The animals were euthanized under deep general anesthesia following completion of experiments.

Animals were randomly assigned to the predefined anesthetic and stimulation groups before recordings. During analysis, full blinding to stimulation modality and anesthetic condition was not feasible because artifact screening, trigger reconstruction, and file organization required examination of the recordings and experimental metadata. However, burst detection was performed using predefined threshold- and duration-based rules, limiting operator-dependent bias compared with manual burst scoring.

The number of animals was kept to the minimum required to obtain repeated valid stimulation trials across the predefined anesthetic and stimulation conditions, in accordance with the principles of replacement, reduction, and refinement.

### 2.2. EEG Electrodes and Surgical Implantation

Anesthesia for electrode implantation was induced with 3.0% *v*/*v* isoflurane in a sealed chamber, followed by an intraperitoneal injection of ketamine (60 mg/kg) and xylazine (5 mg/kg). To maintain anesthesia throughout the procedure, supplemental intramuscular ketamine (60 mg/kg) was administered as required. Surgical depth was ensured by the absence of a tail-pinch response.

After the rat was secured in a stereotactic frame (Neurostar, Tübingen, Germany), the cranial fur was removed using a cordless electric clipper and the exposed skin was disinfected with Betadine. The scalp was then longitudinally incised and the skin and soft tissues were removed to uncover the cranium. FeCl_3_ was applied with a cotton swab to improve adherence of the dental cement.

Custom epidural electrode arrays were constructed using standard 2.54 mm pitch pin header connectors as the primary headstage. Bare silver wires were soldered directly to the connector pins to form individual cortical leads. To improve durability and insulation, the soldered assemblies were coated with acrylic resin while leaving only the distal tips exposed. The exposed silver tips were electrochemically chlorinated in aqueous HCl using a 9 V direct current source to reduce polarization and improve signal transduction at the cortical interface. An electrocorticographic signal was thus acquired, referred to in this study as EEG.

Electrode holes were drilled at the following coordinates relative to bregma: 5 mm anterior, ±2.35 mm lateral; and 6 mm posterior, ±3.35 mm lateral. The electrodes were placed in contact with the dura mater and the connector was secured to the skull with dental cement (UNIFAST Trad, GC Dental Products Corp., Kasugai, Japan). Postoperative care included 0.5 mL intraperitoneal saline for hydration, after which the animals recovered in their home cages for two days before EEG recordings. This implantation strategy and electrode geometry were retained from the previously published photic stimulation model [16].

### 2.3. EEG Recordings and Anesthetic Protocols

EEG recordings were carried out using a BIOPAC MP150 system (BIOPAC Systems, Inc, Goleta, CA, USA) and EEG100C amplifier modules configured at a sampling rate of 1000 Hz, a gain of 10 dB, and a hardware bandpass filter of 1–35 Hz. The implanted headstage was connected to the acquisition system, and a ground electrode was attached at the base of the tail using 1% lithium recording gel (OTE Biomedica, Florence, Italy). A two-channel fronto-occipital bipolar EEG montage was used with AcqKnowledge 4.2, and stimulus triggers were recorded in parallel with the EEG. Cardiac activity was monitored throughout by electrocardiography (ECG).

EEG traces were continuously monitored during experiments, and sedation was titrated to obtain stable EEG burst-suppression—defined as suppression occupying more than 50% of the epoch. For the chloral hydrate group, burst-suppression was induced and maintained with intraperitoneal chloral hydrate titrated at cumulative doses of 400–600 mg/kg. For the isoflurane and sevoflurane groups, anesthesia was delivered by inhalation at concentrations between 2% and 5%.

### 2.4. Stimulation Trials

As in the published photic stimulation model, the effect of stimulation was assessed in 180 s trials comprising three 60 s epochs: baseline (PRE), stimulation (STIM), and recovery (POST) [16].

**Figure 1:**
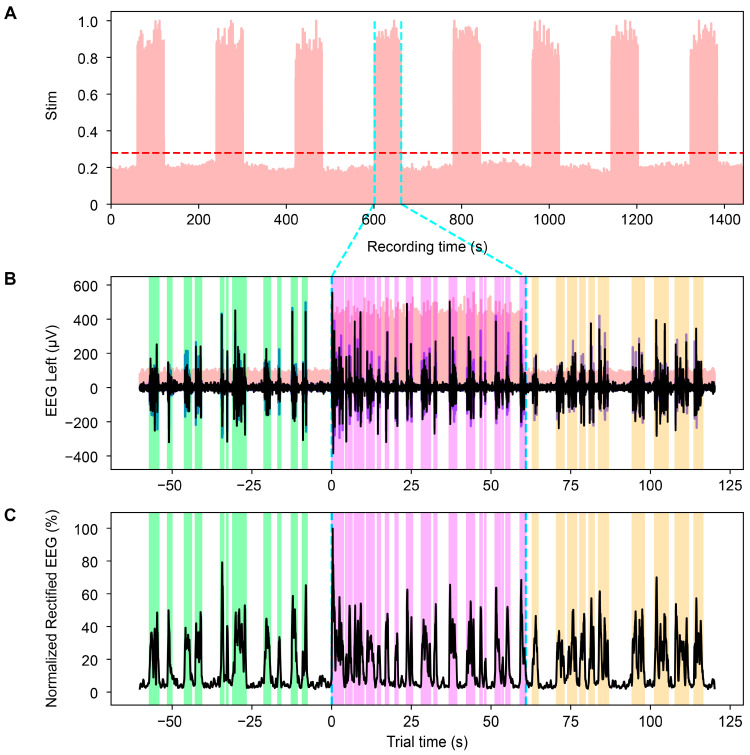
**Representative auditory stimulation recording and within-trial burst**-**suppression analysis.** (**A**) Stimulation channel across the full recording. The horizontal red dashed line marks the trigger threshold, and the vertical cyan dashed lines indicate the representative trial. (**B**) Raw epidural EEG during the selected trial, segmented into PRE (green), STIM (magenta), and POST (orange) epochs. Shaded bands indicate automatically detected generalized bursts, and the stimulus channel is shown in the background in peach orange. (**C**) Normalized rectified EEG from the bilateral channels for the same trial, used for automated burst-suppression analysis.

Auditory stimulation (AUD, Figure 1) consisted of ambient playback of the first minute of Stayin’ Alive by the Bee Gees (RSO, 1977, London, UK). An open-field setup was chosen to approximate the speakers present in an operating room, and volume was kept at 55–60 dB SPL at rat ear level, which would allow unimpaired conversation during surgery. For calibrating the sound delivery, a Type 4939-A-011 free-field microphone, connected to a Type 2610 amplifier, and a Type 4231 sound calibrator (Brüel & Kjær, Virum, Denmark) were used. The speakers were placed at a distance of 50 cm. The background noise in the room was measured to vary between 30- and 40-dB SPL, which was expected for a typical electrophysiology laboratory environment, with no additional equipment noise being present. Although the Wistar rat audiogram is shifted relative to humans and lower song frequencies may have been less audible, the remaining spectral components were within rat auditory capabilities [17]. This stimulus was selected because music is commonly played in operating theaters and because a published survey identified this song as surgeons’ favorite [12].

**Figure 2:**
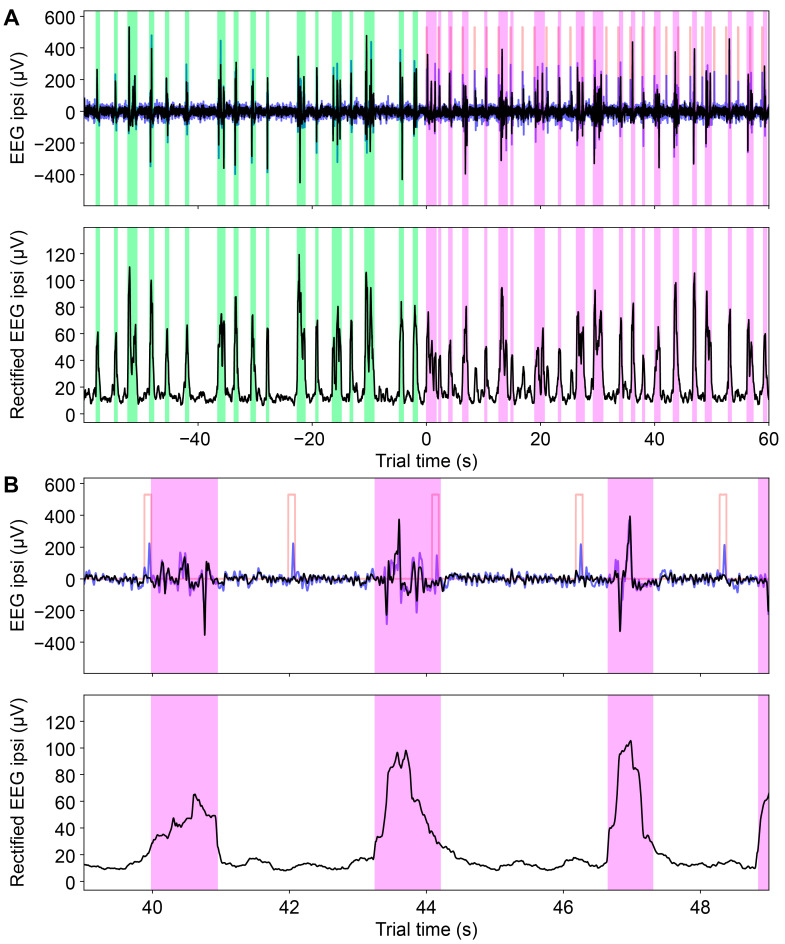
**Representative photic stimulation trial under sevoflurane anesthesia.** (**A**) Raw epidural EEG (top) and rectified EEG (bottom) during PRE (green) and STIM (magenta) epochs. In the raw EEG trace, the channel ipsilateral to the stimulation is shown in black and the contralateral channel in blue. Shaded green and magenta vertical bands indicate detected bursts. The trigger input channel is shown in the background in peach orange. (**B**) Expanded stimulation detail from the same trial. The raw EEG trace (top) again shows the ipsilateral channel in black and the contralateral channel in blue. The rectified EEG (bottom) used for burst identification is the ipsilateral signal. This example illustrates the distinction between the brief stimulus-locked visual evoked potential and the broader burst event used for suppression-based analysis.

Photic stimulation (PHOT) used the established published 0.5 Hz intermittent monocular photic stimulation paradigm (Figure 2) described in a previous study [16].

EEG signals were preprocessed using a zero-phase fourth-order Butterworth bandpass filter (1–35 Hz). Stimulus triggers were detected from the trigger channel by threshold crossing and were used to segment each trial into the PRE, STIM, and POST epochs.

### 2.5. Automated Burst-Suppression Analysis and Metric Extraction

Burst-suppression analysis was performed offline using a custom Python v3.13.9-based GUI tool: BURST (Burst-sUppression Reviewer-Supervised analysis Tool). The logic used by the tool was previously developed for trial-by-trial analysis of rodent burst-suppression EEG recordings [16]. The software imported preprocessed EEG recordings, reconstructed stimulus triggers, and generated a binary burst-suppression representation using threshold- and duration-based rules. It should be noted that, due to the different stimulation patterns between AUD and PHOT, operators could not be blinded to stimulus modality.

The normalized rectified EEG was smoothed and thresholded to obtain a binary burst-suppression signal, which was filtered iteratively using minimum burst duration and minimum suppression duration constraints. The default temporal criteria were a minimum burst duration of 0.35 s, a minimum suppression duration of 0.35 s, and a fixed amplitude threshold of 3 times baseline amplitude, following American Clinical Neurophysiology Society-inspired burst-suppression rules adapted to rodent epidural recordings [1,16]. The same thresholding logic and temporal constraints were applied to both auditory and photic trials. Nevertheless, for photic stimulation trials, burst detection was performed on a reference signal derived from the ipsilateral EEG channel [16], whereas burst detection for auditory stimulation was derived from the average bilateral reference signal.

The trigger input was used to define the epochs, rather than fixed time offsets, in order to account for acquisition variability and preprocessing steps. The pre-stimulation epoch extended from the start of the trial to the first detected trigger, the stimulation epoch extended from the first detected trigger to the last one plus a short post-trigger buffer, and the recovery epoch continued until the end of the trial. For each valid trial, the software exported epoch-specific SR values, epoch durations, trigger counts, and trial validity status into trial-level spreadsheets for downstream analysis. A representative full auditory-stimulation recording, segmented trial, and normalized rectified EEG are shown in Figure 1A–C. A representative photic stimulation trial is shown in Figure 2A,B.

### 2.6. Outcome Measures

Suppression ratio (SR) was calculated for each epoch as the fraction of time spent in suppression [4,16].

The burst-suppression reactivity index (BSRi) was calculated as (SRpre − SRstim)/SRpre. Larger BSRi values therefore reflected a greater stimulation-induced reduction in suppression ratio and thus higher EEG reactivity.

Burst rate and burst duration were analyzed to test the mechanism underlying the SR decrease. Burst rate was expressed as bursts per minute (BPM), calculated for each epoch as burst count divided by epoch duration and multiplied by 60. Burst duration was calculated per epoch as the total epoch duration multiplied by the burst ratio (one minus SR divided by 100) and divided by the burst count.

### 2.7. Statistical Analysis

We included 196 valid 3 min stimulation trials with SRpre ≥ 50% and SRpre < 95% (Table 1). The lower 50% criterion was chosen according to the clinical criteria distinguishing burst-suppression from discontinuous EEG [1], whereas the 95% upper limit was chosen to ensure that at least some bursting activity occurred in PRE.

The primary analysis was performed at the trial level, as justified by the study design. Within each stimulus and anesthetic group, SR across the PRE, STIM, and POST epochs was compared using repeated-measures one-way ANOVA followed by paired Bonferroni-corrected post hoc tests. Group differences in BSRi were tested with one-way ANOVA followed by Bonferroni-corrected pairwise comparisons. To compare auditory and photic stimulation directly, a two-factor ANOVA was fitted for BSRi with anesthesia, stimulation modality, and their interaction as predictors.

Complementary animal-aware analyses were used to evaluate robustness to within-animal clustering [18]. For the stimulation-related SR decrease, data were reshaped to long format and fixed-effects linear models were fitted with SR as the dependent variable and animal identity and epoch as predictors. Animal identity absorbed stable between-animal baseline differences, while epoch effects tested within-animal changes. Cluster-robust standard errors were computed at the animal level [19]. The same animal-aware fixed-effects framework was used for the transient-effect analysis across PRE, STIM, and POST, with planned contrasts for STIM-PRE, POST-PRE, and POST-STIM. For BSRi, repeated trials were modeled using Gaussian generalized estimating equations (GEEs) with exchangeable within-animal correlation and animal identity as the clustering unit [20]. An additive model tested the main effects of stimulation modality and anesthesia, and a second model tested the anesthesia-by-stimulation interaction using joint Wald chi-square tests. Additional mechanistic analyses characterized burst rate and burst duration. BPM during STIM was compared with the trial-wise mean of PRE and POST using paired one-sided Wilcoxon signed-rank tests within each stimulation–anesthetic group, testing the directional alternative that STIM was greater than the mean of PRE and POST; the same comparison was repeated with animal-aware fixed-effects models. Burst duration during STIM was compared with the trial-wise mean of PRE and POST using a paired one-sided Wilcoxon signed-rank test and a corresponding animal-aware fixed-effects model with the same directional alternative, STIM greater than the mean of PRE and POST.

To determine whether the stimulation-related SR decrease was driven primarily by burst recruitment rather than by burst prolongation, a pooled auditory mechanistic analysis compared three linked directional endpoints: SR decrease from PRE to STIM, BPM increase from the mean of PRE and POST to STIM, and burst duration increase from the mean of PRE and POST to STIM. The same contrasts were then tested with animal-aware fixed-effects models containing animal identity and stimulation epoch as predictors.

## 3. Results

### 3.1. Auditory Stimulation Transiently Decreases the SR

**Figure 3:**
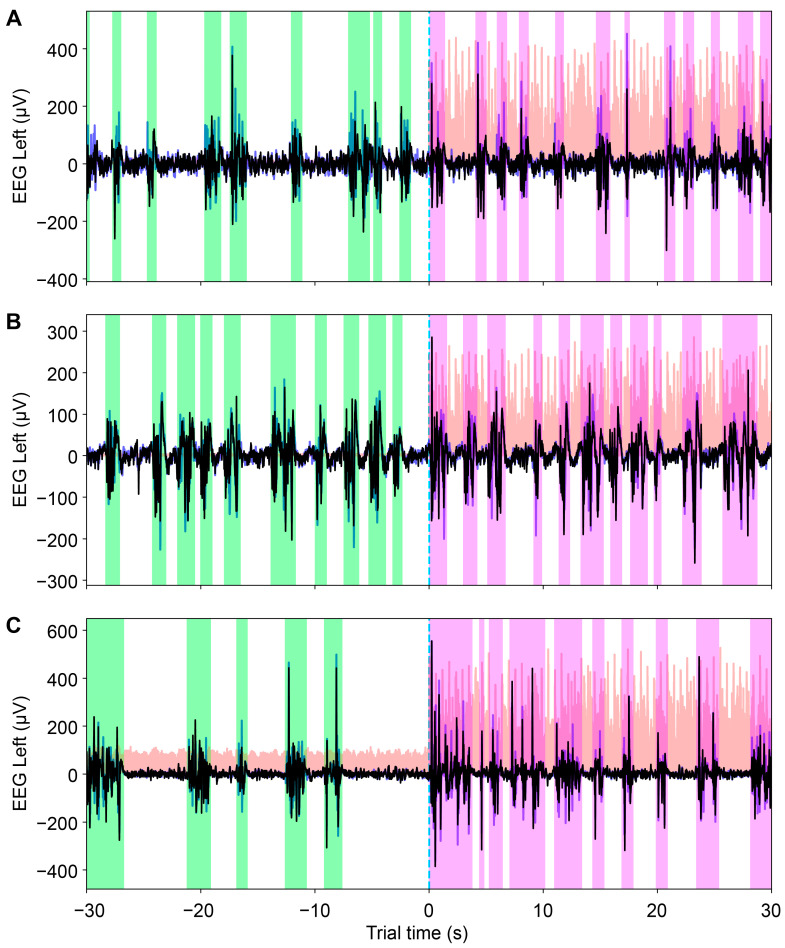
**Representative auditory stimulation trials across anesthetic conditions.** Representative auditory trials recorded during burst-suppression anesthesia under (**A**) sevoflurane, (**B**) chloral hydrate, and (**C**) isoflurane. Raw epidural EEG is shown from 30 s before to 30 s after stimulation onset. Shaded vertical bands indicate automatically detected generalized bursts: green during the pre-stimulation baseline, and magenta shading during the stimulation period. The vertical cyan dashed line marks stimulation onset. The stimulus channel is shown in the background in peach orange.

**Figure 4:**
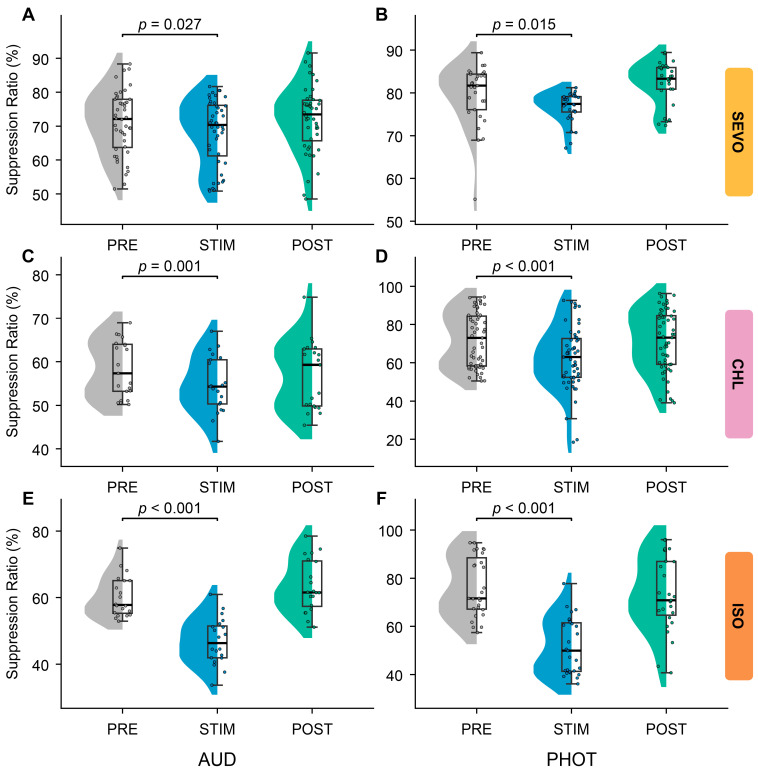
**Trial**-**wise suppression ratio during auditory and photic stimulation across anesthetic conditions.** Suppression ratio (SR) across pre-stimulation baseline (PRE), stimulation (STIM), and post-stimulation recovery (POST) epochs under sevoflurane (SEVO), chloral hydrate (CHL), and isoflurane (ISO) anesthesia for auditory stimulation (AUD) and photic stimulation (PHOT). Panels show (**A**) AUD SEVO, (**B**) PHOT SEVO, (**C**) AUD CHL, (**D**) PHOT CHL, (**E**) AUD ISO, and (**F**) PHOT ISO. Violin plots show the distributions, box plots indicate the median and interquartile range, and points represent individual trials. Horizontal brackets indicate the significant PRE-versus-STIM comparisons within each panel. In both stimulation paradigms, SR decreased during stimulation under all three anesthetic conditions, with the smallest effect under SEVO, an intermediate effect under CHL, and the largest effect under ISO. The magnitude of the reduction was greater in PHOT than in AUD, while the anesthetic ordering remained the same. Abbreviations: AUD, auditory stimulation; PHOT, photic stimulation; SEVO, sevoflurane; CHL, chloral hydrate; ISO, isoflurane.

Auditory stimulation decreased the suppression ratio (SR) under all three anesthetic conditions, and the effect was confined to the stimulation epoch. Representative auditory stimulation trials are shown in Figure 3A–C, and trial-wise SR distributions are summarized in Figure 4A,C,E.

In auditory stimulation trials, mean SR changed from 71.07 ± 1.42 before stimulation to 68.29 ± 1.47 during stimulation and 72.12 ± 1.49 after stimulation under SEVO, from 58.35 ± 1.40 to 54.71 ± 1.41 and 56.87 ± 1.72 under CHL, and from 60.24 ± 1.37 to 46.94 ± 1.56 and 63.44 ± 1.77 under ISO. The within-group effects were significant under SEVO (F_(2,86)_ = 6.39, *p* = 0.0026, partial eta-squared = 0.1294), CHL (F_(2,40)_ = 4.65, *p* = 0.0152, partial eta-squared = 0.1888), and ISO (F_(2,38)_ = 34.06, *p* < 0.0001, partial eta-squared = 0.6419). Bonferroni-corrected PRE-versus-STIM comparisons remained significant in all three groups (SEVO: mean difference 2.78 percentage points, *p* = 0.0272; CHL: mean difference 3.64 percentage points, *p* = 0.0013; ISO: mean difference 13.30 percentage points, *p* < 0.0001).

The recovery epoch was then analyzed explicitly to test whether the auditory effect was transient. In the classical repeated-measures phase analysis across PRE, STIM, and POST, phase was significant for auditory stimulation (AUD; 85 trials from nine animals; F_(2,168)_ = 27.77, *p* = 3.81 × 10^−11^, partial eta-squared = 0.248). Planned Bonferroni-corrected comparisons showed the expected PRE-to-STIM decrease in SR (mean difference 5.47 percentage points, *p* = 1.25 × 10^−7^), no significant difference between PRE and POST (mean difference −0.93 percentage points, *p* = 0.8028), and a significant STIM-to-POST increase in SR (mean difference −6.40 percentage points for STIM minus POST, *p* = 6.18 × 10^−8^). Thus, SR decreased during auditory stimulation and returned toward baseline after stimulation ended.

This transient PRE-STIM-POST pattern is important because it indicates that the auditory effect occurred during active stimulation rather than reflecting a monotonic drift in anesthetic depth, progressive recording instability, or a sustained post-stimulation change.

### 3.2. Auditory Stimulation Increases Burst Count Without Changing Burst Duration

**Figure 5:**
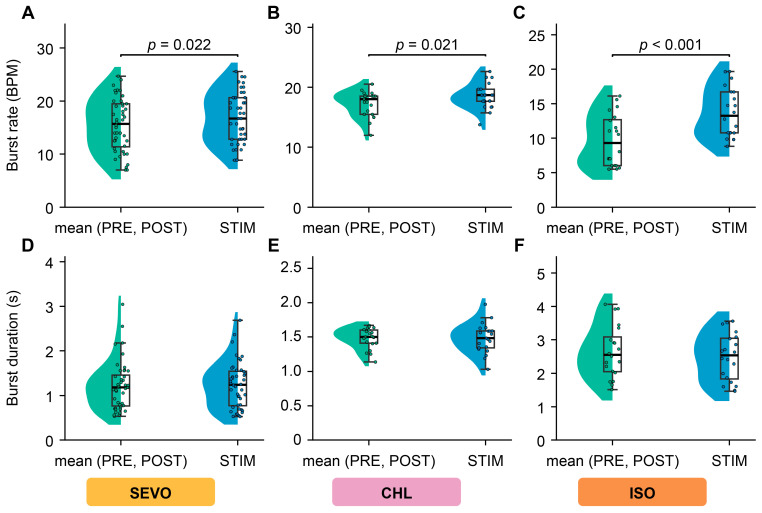
**Burst rate and burst duration during auditory stimulation across anesthetic conditions.** Comparison of burst rate and burst duration during auditory stimulation (AUD) under sevoflurane (SEVO), chloral hydrate (CHL), and isoflurane (ISO) anesthesia. Upper panels show burst rate expressed as bursts per minute (BPM), comparing the stimulation epoch (STIM) with the trial-wise non-stimulation reference defined as the mean of the pre-stimulation and post-stimulation epochs (mean (PRE, POST)). Lower panels show the corresponding burst duration comparisons. Panels: (**A**) burst rate during AUD SEVO; (**B**) burst rate during AUD CHL; (**C**) burst rate during AUD ISO; (**D**) burst duration during AUD SEVO; (**E**) burst duration during AUD CHL; (**F**) burst duration during AUD ISO. Violin plots show the distributions, box plots indicate the median and interquartile range, and points represent individual trials. Auditory stimulation increased burst rate under all three anesthetic conditions, while burst duration did not increase significantly. Statistical annotations correspond to paired one-sided Wilcoxon signed-rank tests for STIM > mean (PRE, POST). These findings support the interpretation that auditory stimulation transiently recruits additional generalized bursts rather than prolonging existing bursts through evoked potential contamination. Abbreviations: AUD, auditory stimulation; BPM, bursts per minute; SEVO, sevoflurane; CHL, chloral hydrate; ISO, isoflurane.

To determine whether the auditory SR decrease reflected recruitment of additional bursts, burst rate was expressed as bursts per minute (BPM) and compared during STIM against the trial-wise mean of PRE and POST (Figure 5A–C). In paired one-sided Wilcoxon signed-rank tests of STIM > mean of PRE and POST, BPM increased during AUD under SEVO (mean difference 1.29 BPM, median 1.05 BPM, *n* = 44, W = 667.0, one-sided *p* = 0.0223), CHL (mean difference 1.21 BPM, median 1.15 BPM, *n* = 21, W = 174.0, one-sided *p* = 0.0210), and ISO (mean difference 3.99 BPM, median 4.02 BPM, *n* = 20, W = 204.0, one-sided *p* < 0.0001).

In contrast, auditory stimulation did not increase burst duration (BD; Figure 5D–F). The paired one-sided Wilcoxon tests for STIM > mean of PRE and POST showed no evidence of burst prolongation under SEVO (mean difference 0.0186 s, *n* = 44, W = 543.0, one-sided *p* = 0.2914), CHL (mean difference 0.0001 s, *n* = 21, W = 120.0, *p* = 0.4459), or ISO (mean difference −0.1664 s, *n* = 20, W = 78.0, *p* = 0.8441). Together, the burst count and burst duration analyses indicate that auditory stimulation reduced SR by recruiting additional bursts, not by prolonging detected bursts.

The pooled AUD mechanistic model supported the same conclusion. Across all AUD trials, SR decreased from PRE to STIM (one-sided Wilcoxon for PRE > STIM: *n* = 85, W = 3077.0, *p* = 2.19 × 10^−8^), BPM increased during STIM relative to the mean of PRE and POST (one-sided Wilcoxon: *n* = 85, W = 2861.0, *p* = 2.97 × 10^−6^), whereas BD did not increase (one-sided Wilcoxon: *n* = 85, W = 1742.0, *p* = 0.6460).

Thus, the observed transient SR decrease was associated with an increase in burst rate and not an increase in burst duration.

### 3.3. Auditory Effects Remain Unchanged When Accounting for Individual Animals

All animal-aware auditory analyses supported the trial-level interpretation. For the overall auditory SR decrease, SR decreased from PRE to STIM after controlling for animal identity (STIM-minus-PRE estimate = −5.47 percentage points, cluster-robust SE = 2.06, 95% CI −9.51 to −1.43, z = −2.65, *p* = 0.0080; 85 trials from nine animals). The animal-aware PRE-STIM-POST phase model confirmed the transient pattern: STIM was lower than PRE (estimate = −5.47 percentage points, 95% CI −9.48 to −1.45, *p* = 0.0076), POST did not differ from PRE (estimate = 0.93 percentage points, 95% CI −0.77 to 2.63, *p* = 0.2830), and POST was higher than STIM (estimate = 6.40 percentage points, 95% CI 1.26 to 11.54, *p* = 0.0147).

The animal-aware burst rate and burst duration analyses gave the same mechanistic interpretation. After absorbing stable animal-specific baseline differences, when comparing STIM with the corresponding trial-wise non-stimulation reference, the fixed-effects model estimated STIM BPM increases of 1.29 BPM for AUD SEVO (95% CI −0.85 to 3.42, one-sided *p* for increase = 0.1188), 1.21 BPM for AUD CHL (95% CI −0.28 to 2.69, one-sided *p* for increase = 0.0558), and 3.99 BPM for AUD ISO (95% CI 3.72 to 4.27, one-sided *p* for increase < 0.0001). In contrast, animal-aware BD models showed no evidence that STIM increased burst duration relative to the non-stimulation reference: 0.0186 s for AUD SEVO (95% CI −0.0594 to 0.0966, one-sided *p* for increase = 0.3203), 0.0000 s for AUD CHL (95% CI −0.1299 to 0.1299, *p* = 0.5000), and −0.1664 s for AUD ISO (95% CI −0.2873 to −0.0455, *p* = 0.9965).

### 3.4. Auditory Stimulation Compared with Visual Stimulation

**Figure 6:**
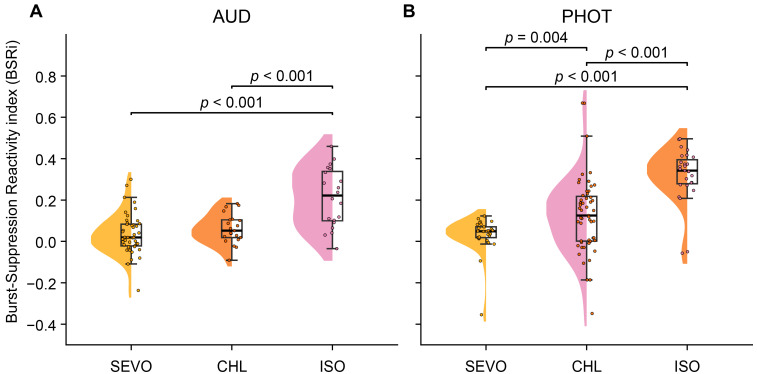
**Burst**-**suppression reactivity index (BSRi) across anesthetic conditions during auditory and photic stimulation.** (**A**) Trial-wise BSRi during auditory stimulation (AUD) under sevoflurane (SEVO), chloral hydrate (CHL), and isoflurane (ISO) anesthesia; (**B**) trial-wise BSRi during photic stimulation (PHOT) under sevoflurane (SEVO), chloral hydrate (CHL), and isoflurane (ISO) anesthesia. Violin plots show the distributions, box plots indicate the median and interquartile range, and points represent individual trials. In panel (**A**), BSRi differed significantly between ISO and SEVO (*p* < 0.001) and between ISO and CHL (*p* < 0.001), while no significant difference was found between SEVO and CHL. In panel (**B**), BSRi differed significantly between CHL and SEVO (*p* = 0.004), between ISO and CHL (*p* < 0.001), and between ISO and SEVO (*p* < 0.001). Comparisons are shown only within each panel. Abbreviations: SEVO, sevoflurane; CHL, chloral hydrate; ISO, isoflurane.

Comparisons of BSRi were carried out to control for differences in pre-stimulation suppression depth (Figure 6A,B). For both AUD and PHOT, BSRi increased in the order SEVO < CHL < ISO. Furthermore, reactivity was larger overall for PHOT than for AUD.

Burst-suppression reactivity to AUD depended on the anesthetic used, increasing in the order SEVO < CHL < ISO. Mean auditory BSRi was 0.0357 ± 0.0149 under SEVO, 0.0607 ± 0.0157 under CHL, and 0.2129 ± 0.0318 under ISO. Group differences were significant (F_(2,82)_ = 20.24, *p* < 0.0001, eta-squared = 0.3305). Bonferroni-corrected pairwise testing showed no significant difference between SEVO and CHL (*p* = 0.7543), whereas ISO differed from both SEVO (*p* = 0.0001) and CHL (*p* = 0.0006).

PHOT showed the same anesthetic ordering as AUD but a generally larger effect. Mean PHOT BSRi was 0.0315 ± 0.0159 under SEVO, 0.1291 ± 0.0248 under CHL, and 0.3203 ± 0.0254 under ISO. Group differences were significant (F_(2,108)_ = 26.32, *p* < 0.0001, eta-squared = 0.3277), and all three PHOT groups differed significantly (SEVO vs. CHL *p* = 0.0043; SEVO vs. ISO *p* < 0.0001; CHL vs. ISO *p* < 0.0001).

Direct AUD-PHOT comparison of BSRi (Figure 6A,B) showed no difference under SEVO (*p* = 1.0000), a non-significant trend toward larger PHOT reactivity under CHL (*p* = 0.0682), and significantly larger PHOT reactivity under ISO (*p* = 0.0354). Across all trials, two-factor ANOVA showed a significant main effect of anesthesia (F_(2,192)_ = 43.09, *p* < 0.0001) and a significant main effect of stimulation modality (F_(1,192)_ = 6.03, *p* = 0.0150), with no significant interaction between anesthesia and stimulation (F_(2,190)_ = 2.64, *p* = 0.0743).

Thus, visual stimulation produced stronger burst-suppression reactivity overall, but the anesthetic ordering was preserved across auditory and visual stimulation paradigms.

### 3.5. Comparison with Photic Stimulation Remained Unchanged When Accounting for Individual Animals

For BSRi, the animal-clustered GEE model preserved the same anesthetic ordering, with BSRi higher under ISO than CHL (estimate = 0.1559, SE = 0.0375, 95% CI 0.0823 to 0.2294, *p* < 0.0001) and lower under SEVO than CHL (estimate = −0.0726, SE = 0.0369, 95% CI −0.1450 to −0.0003, *p* = 0.0490). The animal-clustered GEE analysis also preserved the same qualitative modality interpretation: PHOT produced larger BSRi than AUD overall (PHOT versus AUD estimate = 0.0592, SE = 0.0302, 95% CI 0.0001 to 0.1183, *p* = 0.0498), while the anesthesia-by-stimulation interaction was not significant (joint Wald chi-square = 3.47, df = 2, *p* = 0.1765).

## 4. Discussion

This study examined whether ambient music alters the suppression ratio (SR) during targeted burst-suppression anesthesia in adult male Wistar rats. Using an established SR-based rat burst-suppression framework we found that auditory stimulation transiently reduced SR under sevoflurane, chloral hydrate, and isoflurane. The decrease in SR was associated with increased burst rate without increased burst duration. The BSRi changes depended on the anesthetic type—an effect mirrored by photic stimulation. Our experimental data suggest that targeted suppression ratio reflects both anesthetic depth and the level of ambient stimulation. Ambient sound in the operating theater or intensive care settings could influence EEG-based measures used for anesthesia monitoring.

### 4.1. SR Detection

For burst detection during targeted anesthetic burst-suppression, here we used a simple supervised amplitude threshold method to preserve translational relevance to current clinical practice, in agreement with our previous rodent studies on the same model [16]. Nevertheless, automatic characterization of burst-suppression patterns remains a challenging technical problem as not all EEG amplitude changes can be considered bursts. Artificial Intelligence (AI)-based methods for quantifying burst-suppression patterns have evolved from simple threshold-based segmentation algorithms to advanced machine learning (ML) and deep learning frameworks capable of extracting clinically relevant biomarkers from large and heterogeneous datasets.

Traditional computational approaches focused on deterministic signal-processing methods based on local variance, amplitude thresholds, and suppression duration to estimate indices such as the burst-suppression ratio (BSR) or burst-suppression probability (BSP) [21,22,23]. These methods provided interpretable and computationally efficient bedside monitoring tools but were limited by sensitivity to artifacts and inter-patient variability. More recent AI-based approaches incorporate supervised and unsupervised ML models using handcrafted temporal, spectral, and nonlinear EEG features, including band power, entropy, line length, interburst interval duration, and connectivity metrics. Random forest, support vector machine, CatBoost [24] and gradient boosting models [25] have demonstrated high performance for detecting burst-suppression and predicting neurological recovery after cardiac arrest.

Deep learning methods further extend this paradigm by learning directly from raw EEG signals or time–frequency representations without manual feature engineering. Convolutional neural networks, recurrent neural networks, and hybrid architectures have been used to characterize burst morphology and long-range temporal dependencies in EEG recordings from intensive care, neonatal monitoring, and post-anoxic coma cohorts. Supervised deep learning algorithms have achieved high accuracy for burst and interburst segmentation, with external validation across independent cohorts [26,27]. Deep learning applied to auditory EEG responses after cardiac arrest could predict awakening and survival, with model performance influenced by EEG reactivity and background burst-suppression patterns [28]. Similarly, multiscale deep neural networks captured both short- and long-timescale EEG dynamics for neurological prognostication in comatose patients [29]. However, major limitations remain, including small and heterogeneous datasets, inter-rater variability in burst-suppression annotation, species-specific EEG morphology, limited external validation, and the need for low-latency real-time processing.

### 4.2. Auditory Stimulation by Ambient Music Transiently Decreases the SR

The main finding of this study is that, under targeted burst-suppression, auditory stimulation by ambient music causes a decrease in SR across all anesthetic conditions tested: SEVO, CHL and ISO (Figure 3 and Figure 4). After stimulation, the SR recovered to the pre-stimulation baseline. The transient nature of this effect argues against a simple monotonic drift in anesthetic depth, progressive recording instability, or a sustained post-stimulation shift in the burst-suppression state. Instead, the temporal profile reflects changes due to the presence of the auditory stimulus.

Auditory stimulation in rats evokes brainstem auditory evoked potentials, which are short-latency electrophysiological responses generated along the auditory pathway following acoustic stimulation [30], followed within 400 ms by event-related cortical potentials [31,32]. It is thus theoretically possible that the decrease in SR observed in our study reflected contamination by evoked potentials and not a change in bursting. We do not think that this was the case for several reasons. First, the montage used in our study (fronto-occipital) was far from the local sources of brainstem auditory potentials. Second, for auditory stimulation trials, burst segmentation was based on generalized bursts detected from the normalized rectified bilateral EEG signal, which further favors global cortical activity [8,33]. Third, and most importantly, the burst duration during stimulation did not increase (Figure 5D–F), as could have hypothetically occurred by the addition of evoked activity prior to the burst, as in the case of photic stimulation [11,16].

### 4.3. Auditory Stimulation Transiently Increases the Burst Rate

To further understand the mechanisms underlying the decrease in SR during auditory stimulation, we investigated the associated changes in bursting. We found that auditory stimulation is associated with a transient increase in the number of bursts across all three anesthetic conditions (Figure 5A–C). A comparable increase in burst rate without an increase in burst duration also occurred during photic stimulation, in agreement with previous experimental [16,34] and clinical [11] reports. Taken together, these data suggest that auditory stimulation increased the probability of recruiting generalized bursts during the suppression periods. A similar interpretation was proposed for intraoperative somatosensory stimulation, where SR decreased transiently while burst duration remained approximately unchanged [10]; however, due to the nature of the auditory stimulation employed (naturalistic cues versus discrete artificial clicks), here we could not carry out further per-stimulus studies.

The finding that auditory stimulation evoked bursts is consistent with the broader view that burst-suppression is not a purely passive EEG state. Experimental and clinical studies have shown that external stimulation can trigger or facilitate cortical activity during burst-suppression, indicating that the balance between bursting and suppression can remain sensitive to sensory input even under deep anesthesia [2,3,9,10,11]. The current study extends this concept to a standardized auditory perturbation applied during a controlled rat burst-suppression preparation.

### 4.4. The Auditory Reactivity Was Comparable to Photic Reactivity Across Different Anesthetics

We found that the magnitude of burst-suppression reactivity during auditory stimulation, as measured by BSRi, differed between anesthetic conditions, increasing in the order SEVO < CHL < ISO (Figure 6A). Intriguingly, the same order of BSRi changes was observed during photic stimulation (Figure 6B). Taken together, these data support the conclusion that the magnitude of burst-suppression reactivity to external stimulation depends on the anesthetic used. The comparable anesthetic ordering in this context is consistent with the broader concept that burst-suppression architecture differs across anesthetic agents [35,36].

Auditory evoked EEG responses also remain measurable under anesthesia and are influenced by anesthetic conditions [14,15]. The smaller magnitude of reactivity under SEVO could not be attributed to the absence of evoked responses, as indicated by photic stimulation results (Figure 2B). More likely, it reflected the refractory behavior of burst-suppression networks proposed by Amzica and further developed in related burst-suppression studies [2,3,9], although investigating anesthetic-dependent mechanisms remains beyond the scope of this study.

An unexpected finding in this study was that burst-suppression reactivity measured by BSRi was consistently smaller for auditory stimulation than for photic stimulation. Although the effect of stimulation intensity was not directly addressed in this study, it was previously proposed that under burst-suppression the brain is hyperreactive, and even the smallest stimuli could evoke bursts [9]. It is thus possible that the differences reflect a modality-dependent effect, in addition to the anesthesia-dependent effect.

### 4.5. Methodological Limitations

The study was carried out on healthy Wistar rats of the same age and sex. In order to limit the use of experimental animals, we carried out a repeated trial design statistically powered based on previous studies using the same model [16], ensuring at least two animals per group. This was sufficient to address the transient changes in SR and burst rate in paired comparisons per trial. Furthermore, the differences remained robust when accounting for animal identity using a fixed-effects linear model supporting the main objective of the study. Our data also suggest that both the anesthetic agent and the stimulation modality influence the burst-suppression reactivity magnitude. Although the auditory and photic datasets were comparable in size, and the magnitude of BSRi changes remained robust after accounting for within-animal clustering repeated trials from the same animal cannot replace independent biological replication across conditions [18]. Further studies should be carried out to characterize the individual factors affecting burst-suppression reactivity at the population level.

The photic stimulation paradigm is relevant here because it provides an established methodological benchmark. The previously published 0.5 Hz photic stimulation model demonstrated robust SR-based burst-suppression reactivity in rats [16], allowing the auditory effect to be interpreted against a sensory paradigm already known to modulate burst-suppression. The same thresholding logic and temporal constraints were applied to both auditory and photic trials. Consistent with our previously published photic stimulation model, in this study during photic stimulation trials bursts were detected on a reference signal derived from the ipsilateral EEG channel to minimize contamination by stimulus-locked contralateral visual evoked potentials [16]. In contrast, bursts during auditory stimulation trials were derived from a bilateral reference signal. This choice was made because the auditory stimulus was delivered as an open-field sound rather than as a strictly lateralized monaural stimulus, and auditory responses during anesthesia are expected to involve bilateral cortical processing to varying degrees [14,15,37]. This methodological difference is physiologically justified, but it limits a strictly quantitative comparison of auditory and photic BSRi values. The photic condition should therefore be interpreted as a methodological comparator for direction, ordering, and relative magnitude of reactivity, not as a fully matched modality-control experiment.

### 4.6. Translational Considerations

The current experimental study was motivated by the need to assess whether ambient music affects the SR commonly used to monitor the depth of burst-suppression during targeted deep anesthesia or medically induced coma [4,5,6,7]. Traditionally, these monitors were not presumed to be affected by auditory stimulation [38]. The simple scenario that ambient music could alter the SR-based EEG metrics could have implications for automated anesthesia tracking and control systems, including rodent closed-loop burst-suppression control and BIS-guided anesthesia delivery [22,39,40,41,42,43,44] across different anesthetic conditions [23].

In our study, the auditory paradigm itself was limited to one stimulus and one sound intensity range. The first minute of Stayin’ Alive was selected as a standardized, spectro-temporally complex sound with contextual relevance to operating room music, but the present data do not determine whether the effect depends on rhythm, intensity, spectral content, temporal structure, or stimulus familiarity. Rats also do not perceive this stimulus in the same way as humans. Their hearing range extends further into high frequencies, and their frequency sensitivity differs across the audible band [17,45,46]. Therefore, the auditory stimulus should not be interpreted as a model of the human subjective experience of intraoperative music. It is better viewed as a standardized auditory perturbation applied to a mammalian burst-suppression preparation with preserved auditory responsiveness [14,15,37,47].

It should also be noted that the translational scope is limited by the recording configuration and species. The recordings were epidural rat cortical signals, referred to as EEG for consistency with the burst-suppression monitoring literature, but they are not equivalent to human scalp EEG or processed clinical indices such as BIS. Epidural recordings are influenced by electrode geometry, impedance, interelectrode distance, local cortical signal properties, and the rules used to extract binary burst-suppression metrics [1,8]. The present study minimized these sources of variability by using the same electrode placement, acquisition setup, preprocessing logic, and BURST-based pipeline used in the published rat photic stimulation model [16].

## 5. Conclusions

We provide experimental evidence that, in a rodent model, ambient music decreases the suppression ratio during targeted burst-suppression achieved by several general anesthetics. This hypothesis-generating study provides proof-of-concept for future controlled randomized clinical trials investigating whether the level of the environmental sound should be accounted for when suppression ratio is used as a monitoring or control target under various anesthetics.

## Figures and Tables

**Table 1:** Included trials and represented animals by stimulus paradigm and anesthetic condition.

Stimulus Paradigm	Anesthetic Condition	Included Trials, *n*	Animals Represented, *n*
Auditory stimulation (AUD)	Sevoflurane (SEVO)	44	4
Auditory stimulation (AUD)	Chloral hydrate (CHL)	21	3
Auditory stimulation (AUD)	Isoflurane (ISO)	20	2
Photic stimulation (PHOT)	Sevoflurane (SEVO)	29	2
Photic stimulation (PHOT)	Chloral hydrate (CHL)	55	2
Photic stimulation (PHOT)	Isoflurane (ISO)	27	2

AUD, auditory stimulation; PHOT, intermittent photic stimulation; SEVO, sevoflurane; CHL, chloral hydrate; ISO, isoflurane.

## Data Availability

Data is available upon reasonable request to the corresponding author.

## Abbreviations

The following abbreviations are used in this manuscript:

EEG	Electroencephalogram
SR	Suppression ratio
BSRi	Burst-suppression reactivity index
BIS	Bispectral Index
AUD	Auditory stimulation
PHOT	Photic stimulation
SEVO	Sevoflurane
CHL	Chloral hydrate
ISO	Isoflurane
BURST	Burst-sUppression Reviewer-Supervised analysis Tool
PRE	Pre-stimulation baseline epoch
STIM	Stimulation epoch
POST	Post-stimulation recovery epoch
BPM	Bursts per minute
BD	Burst duration
GEEs	Generalized estimating equations
